# A Novel Approach for Effective Alteration of Morphological Features of Polyaniline through Interfacial Polymerization for Versatile Applications

**DOI:** 10.3390/nano10122404

**Published:** 2020-11-30

**Authors:** Kalyan Vaid, Jasmeen Dhiman, Suresh Kumar, Ki-Hyun Kim, Vanish Kumar

**Affiliations:** 1 National Agri-Food Biotechnology Institute (NABI), S.A.S. Nagar, Punjab 140306, India; vaidkalyan@gmail.com (K.V.); jasmeendhiman10@gmail.com (J.D.); 2 Centre for Nanoscience and Nanotechnology, Panjab University, Chandigarh 160014, India; 3 Department of Applied Sciences, UIET, Panjab University, Chandigarh 160014, India; skphysicsnano@gmail.com; 4 Department of Civil and Environmental Engineering, Hanyang University, 222 Wangsimni-ro, Seoul 04763, Korea

**Keywords:** polyaniline, gold nanoparticles, functionalization, interfacial polymerization, morphology

## Abstract

Morphological characteristics of any nanomaterial are critical in defining its properties. In this context, a method to control morphological parameters of polyaniline (PANI) has been investigated by producing its composite with gold nanoparticles (AuNPs). Herein, we report for the first time the successful control on the physical/chemical properties of PANI composites synthesized via interfacial polymerization through functionalization of its AuNP composite component with citrate, ascorbate, glutathione (GSH), and cetyl trimethyl ammonium bromide (CTAB). A significant difference in the polymerization pattern, morphologies, and electrical properties was recognized in these composites according to the functionality of the modified AuNPs. The obtained composites of AuNPs/PANI exhibited highly diverse morphologies (e.g., nodule, hollow hemisphere, flake, and spider-web galaxy type) and electrical characteristics according to functionalization. Hence, this study is expected to offer better insight into control of the polymerization pattern of AuNP/PANI composites and their associated properties.

## 1. Introduction

Polyaniline (PANI) is one of the most explored conductive polymers used for diverse technological applications [1]. Because of its unique chemical and structural properties, PANI has been readily employed in nanoelectronic devices, electron field emitters, data storage, actuators, rechargeable batteries, supercapacitors, electromagnetic interference shielding, catalysis, gas sensing, and sensors [2,3]. In general, PANI exists in three different oxidation states, namely, leucoemeraldine, emeraldine, and pernigraniline [4]. Of these oxidation forms, emeraldine PANI is of special interest due to its conductive nature. The use of metal nanoparticles (NPs) with PANI has been demonstrated for considerable enhancement of its innate characteristics (e.g., electrical conductivity and surface area) [5,6]. Gold nanoparticles (AuNPs) are among the more promising materials that have been explored to enhance the properties of PANI. AuNPs used during the synthesis of PANI have been highly useful in elevating the yield (e.g., from 0.01% to 10%) of PANI [7].

A number of methods have been employed to prepare PANI and its composites with AuNPs. Among them, interfacial polymerization has shown extraordinary potential, especially for the formation of conductive PANI-based products [8]. Generally, an amalgam of AuNPs with PANI displays a synergistic effect by yielding a composite material with enhanced electrochemical/electro-catalytic and sensing capabilities [9,10]. AuNP/PANI composites have also been found beneficial in diverse technological applications, e.g., in development of room temperature resistive sensors for gases/vapors [11], as memory devices in the field of organic electronics [1], and in environmental/biomedical applications [11].

A special control over the morphology of PANI is of longstanding interest for the expansion of its applicability in diverse fields [12]. A large number of strategies have already been exploited to endow PANI with special characteristics such as electrospinning, interfacial polymerization, and template-based synthesis [13]. However, it is not yet possible to directly synthesize PANI-based materials with desired/controlled morphology through the alteration of the functionality of their composite component (e.g., AuNPs). Herein, we have for the first time explored the applicability of an interfacial polymerization approach to control the structural parameters of PANI nanofibers (NFs) by forming their composites with functionalized AuNPs. We have achieved controlled and desired morphology of PANI in the form of AuNP/PANI composite by varying the capping of AuNPs. Four types of functionalization including (1) citrate, (2) ascorbate, (3) glutathione (GSH), and (4) cetyl trimethyl ammonium bromide (CTAB) have been applied to first cap AuNPs, which were then subjected to the final fabrication of AuNP/PANI composites in four distinctive morphologies of nodular structures, hollow hemispherical, flakes, and spider web galaxy type, respectively. Each of these four distinctive structures vary in fiber size and electronic properties. To the best of our knowledge, no other attempt has been made to date to explore the effects of different functionalization of AuNPs on the related properties of PANI. The diversification of morphological properties of PANI composites is expected to contribute significantly to the progressive development of their technological applications in fields such as energy storage, sensing, pollutant removal, medical imaging, and as delivery vehicles.

## 2. Materials and Methods

Chloroauric acid (HAuCl_4_), aniline, and chloroform were purchased from Central Drug House (CDH) (P) Ltd, Gujrat, India. Trisodium citrate, sodium borohydride, CTAB, ascorbic acid, and ammonium persulfate were procured from Sigma-Aldrich, Co., St. Louis, MO, USA. GSH was obtained from Sisco Research Laboratories (SRL) Pvt Ltd, Mumbai, India. Sodium hydroxide and hydrochloric acid were purchased from Green Genome (P), Ltd., Delhi, India. All the purchased chemicals were of analytical grade. All aqueous solutions were prepared using Milli-Q (MQ; Millipore SAS, Molsheim, France) water with resistivity of 18 MΩ cm.

To begin with, we prepared citrate-, CTAB-, GSH-, and ascorbate- capped AuNPs for the formation of AuNP/PANI composites. The corresponding synthesis methods employed for the functionalized AuNPs were adopted from Turkevich, et al. [14], Pannico, et al. [15], Chai, et al. [16] and Rastogi, et al. [17], respectively. Synthesis of PANI and its composites was achieved by adopting and modifying the approach used by Kumar, Mahajan, Bhatnagar and Kaur [8]. We briefly describe all the adopted methodologies here. The detailed synthetic methods can be accessed in the cited articles.

Citrate-capped AuNPs were obtained by the reduction of chloroauric acid with sodium citrate [14]. The sodium citrate acts as a reducing and stabilizing agent for HAuCl_4_ and AuNPs, respectively. The CTAB-capped AuNPs were synthesized using the 3-step method [15]. In Step 1, equal volumes of 0.5 mM HAuCl_4_ and trisodium citrate were stirred for 10 min followed by dropwise addition of ice-cold aqueous solution of 100 mM sodium borohydride with continuous stirring to prepare seed solution. In Step 2, growth solution was prepared by dissolving 1.5 g of CTAB solid powder in 100 mL of 0.5 mM chloroauric acid aqueous solution. The resulting solution was heated at 50 °C with continuous stirring until clear solution was obtained. In Step 3, seed growth was allowed to take place. During this step, 100 mM ascorbic acid was added into the growth solution with continuous stirring, followed by dropwise addition of seed solution. Afterwards, the obtained solution was kept stirring at 350–400 rpm for 15 min. After standing for 30 min, the prepared CTAB-capped AuNPs were washed with MQ water (twice) at 10,000 rpm for 30 min [15].

GSH-capped AuNPs were synthesized by mixing aqueous solutions of 25 mM chloroauric acid and 19 mM GSH. The pH of the solution was adjusted to 8.0 with freshly prepared 1 M sodium hydroxide, followed by addition of 52.86 mM sodium borohydride under continuously stirring conditions. The reaction mixture was kept overnight at room temperature followed by centrifugation at 6000 rpm for 10 min to remove free GSH molecules [16]. For synthesis of the ascorbate-capped AuNPs, the pH of 39 mM aqueous solution of ascorbic acid was adjusted to 7–8 using sodium hydroxide [17]. Red wine-colored AuNPs were obtained by adding and stirring 1 M aqueous solution of chloroauric acid into the aforementioned ascorbic acid solution [17].

Polyaniline (PANI) and its composites with capped AuNPs were prepared using interfacial polymerization under controlled conditions. We used chloroform as the organic phase and 1 M HCl as the aqueous phase. Before forming the interface, 7 µL of aniline (monomer for polyaniline) were dissolved in 10 mL of the organic phase [8]. Likewise, 2 mg of ammonium persulfate was added in 10 mL of 1 M HCl. The PANI was synthesized by making an equivolume interface between organic (bottom layer) and aqueous phase (upper layer) in a reaction vessel [8]. Note that care must be taken while pouring aqueous phase onto the organic phase for effective interface formation. The contents were kept undisturbed overnight for complete polymerization of aniline to make PANI fibers. In the case of AuNP/PANI composites, AuNPs capped with citrate, CTAB, GSH, and ascorbate were also added to separate aqueous phases prior to formation of the interface in respective reaction vessels. All the other steps used for the preparation of AuNP/PANI composites were the same as used for PANI synthesis. The dark green colored fibrous material product was isolated and washed using deionized water, methanol, acetone, and diethyl ether to remove unused chemicals [8]. The equal amounts of acid were used in each case to maintain similar conditions for all the samples. It should be noted that any variation in pH could lead to the alteration in doped or de-doped states of PANI to impart various characteristics.

After completion of the reaction, the synthesized PANI and its composites with AuNPs were characterized using ultraviolet–visible (UV-Vis) spectroscopy (Shimadzu UV-2700; Shimadzu Corporation, Kyoto, Japan), Fourier-transform infrared spectroscopy (FTIR) (PerkinElmer, Llantrisant, UK), field emission-scanning electron microscope (FE-SEM) (Hitachi SU 8010; Hitachi Ltd., Krefeld, Germany), and transmission electron microscopy (TEM) (Jeol JEM-1400 EM; JEOL Inc, Peabody, MA, USA).

The electrochemical characteristics of prepared materials were examined on gold (Au) screen printed electrodes (SPE) using Metrohm Autolab M204 workstation (Metrohm Autolab B.V., Utrecht, Netherlands). 50 µL of the test materials (e.g., PANI and AuNP/PANI) were drop casted and dried (at room temperature) on Au-SPE to record the voltammogram. The voltammograms were recorded using 50 µL of 0.5 M H_2_SO_4_ as electrolyte solution. The upper and lower vertex potential was set to 0.8 V and −0.2 V, respectively. The start and stop potentials were 0 V. The data were recorded in 10 scans with scan rate and step size of 0.1 V/s and 0.00244 V, respectively.

## 3. Results and Discussion

Herein, citrate-, GSH-, ascorbate, and CTAB-capped AuNPs were formed by chemical synthesis methods. The modified AuNPs were further used to prepare corresponding AuNP/PANI composites via interfacial polymerization. The composite materials and pure PANI (prepared via interfacial polymerization) were subjected to several analysis techniques to interrogate their morphological and electronic characteristics.

The synthesized PANI and its composite structures with citrate, ascorbate, and CTAB-functionalized AuNP exhibited dark green color, whereas that for GSH-AuNP/PANI composite was brown (Figure S2). It is worth mentioning that the green color of PANI and AuNP/PANI composites was mainly due to the presence of emeraldine salt form of PANI, while the brown color in GSH-AuNP/PANI should reflect the presence of pernigraniline form of PANI [18]. In order to validate the control over synthesized PANI and AuNP/PANI composite, in terms of physical/chemical characteristics, the synthesis (followed by characterization) of these materials was performed several times. Interestingly, similar results were obtained each time to indicate a good hold over the controlled synthesis of PANI and its composites.

### 3.1. UV-Vis Characterization of PANI and its AuNP Composites

**Figure 1:**
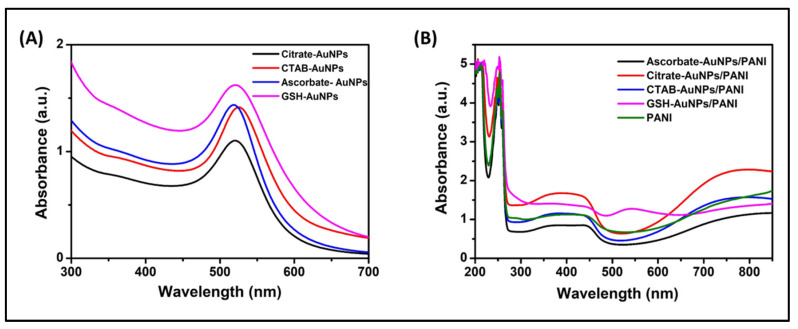
Ultraviolet–visible **(**UV-Vis) spectra of (**A**) AuNPs capped with different agents and (**B**) PANI and AuNP/PANI composites.

The PANI and AuNP/PANI composites were examined with UV-Vis spectroscopy for electronic transitions. In the cases of citrate-, GSH-, and ascorbate-capped AuNPs, strong localized plasmon resonance (LSPR) absorption peaks were observed at 521 nm. The analogous peak was observed at 526 nm for CTAB-capped AuNPs (Figure 1A). These peaks can be ascribed to LSPR of AuNPs. The collective oscillations of electrons are mainly postulated for the LSPR, due to which AuNPs display a strong absorbance band in the visible region ranging between 500 and 600 nm. Note that the shape and size of the AuNPs are critical parameters which give rise to the LSPR absorbance maximum [15,16,17,19].

In the case of PANI and three out of four of its composites (citrate-AuNPs/PANI, ascorbate-AuNPs/PANI, and CTAB-AuNPs/PANI), two main peaks were observed at 350 and 440 nm in UV-Vis spectra (Figure 1B), which correlate with the emeraldine salt of PANI [8,20]. These two peaks amalgamate to form a flat single band corresponding to the presence of cation radicals along with a π–π* transition in the benzoid ring and a polaron transition in the quinoid ring [9,20,21,22]. Another peak obtained in the range of 750–800 nm can be attributed to the bipolar transition (exciton) of quinoid ring due to charge transfer from adjacent benzoid ring. The peaks at 440 and 750–800 nm may represent the effects of protonation by polyaniline chains [9,21]. These peaks are all characteristic of doped and conducting forms of PANI. A few other peaks were also spotted for PANI and AuNP/PANI composites ranging from 248 to 260 nm, indicating the presence of anilinium ions [8]. 

Interestingly, in the case of GSH-AuNP/PANI spectrum, the pattern was distinguished from all other composites described above in that there was an additional absorbance peak (along with above-mentioned peaks) at 550 nm. This can be attributed to the pernigraniline form of PANI, as also indicated by the brown colored appearance of GSH-AuNP/PANI composite [23]. Consequently, it can be concluded that the GSH-AuNPs favored the growth of pernigraniline as well as emeraldine form of PANI. In contrast, the growth of emeraldine salt form of PANI was apparent for citrate-AuNPs, ascorbate-AuNPs, and CTAB-AuNPs. Note that the peak at 550 nm could have been accredited to the SPR of AuNPs. However, the color of GSH-AuNP/PANI composite along with the absence of respective peak in other AuNP/PANI composites was supportive of the presence of pernigraniline form of PANI.

### 3.2. FTIR Characterization of PANI and AuNP/PANI Composites

**Figure 2:**
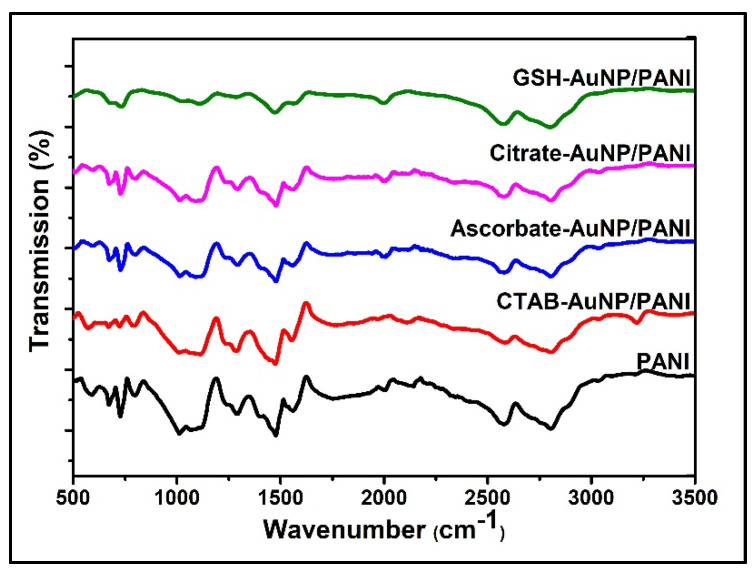
FTIR spectra of PANI and capped-AuNP/PANI composites.

The presence of functional groups in synthesized nanostructures (PANI and AuNP/PANI composites) and their chemical structures were studied by FTIR (Figure 2). Spectra ranging from 3500 to 500 cm^−1^ were recorded and analyzed for all the samples (Table S1). Similar characteristic peaks were observed for PANI and all its composites except GSH-AuNP/PANI. In all samples, characteristic peaks at 1475–1480 and 1560 cm^−1^ were obtained for C=C stretching of benzenoid ring in –NH–B–NH– units and quinoid ring polaronic structures (–B–NH^+^–), respectively [2,3,5,9,20,24]. Likewise, the peak at 1400 cm^−1^ can be ascribed to phenazine type segments, formed due to oxidative intramolecular cyclization of branched oligoaniline (OANI) and polyaniline (PANI), as found in PANI, citrate-AuNP/PANI, and ascorbate-AuNP/PANI [5]. The C–H stretching of aromatic amine can be associated with the peak at 1291–1295 cm^−1^ in all cases [3,25]. Another peak at 1233 cm^−1^, consistent in all spectra except in that of GSH-AuNP/PANI, can be assigned to C–N stretching vibrations in benzenoid unit, representing the conductive form of PANI obtained in pristine form as well as in composite forms including citrate-, ascorbate-, and CTAB-AuNP/PANI [3,5,9]. The peak at 1124 cm^−1^ was assigned to N–H stretching in PANI and AuNP/PANI composites; the lowest intensity of the peak was seen from GSH-AuNP/PANI [3,5]. The peak at 1077 cm^−1^ was sharp in all the cases, whereas it was blunt in case of GSH-AuNP/PANI [26]. Imine deformation (C–N–C bending) and quinone ring deformation were observed at 730 and 799 cm^−1^, respectively, in all cases [9,24]. The peaks at 600 and 1026 cm^−1^ were associated with in-plane deformation vibrations of aniline groups of bipolaronic structure of PANI and in-plane bending of C–H of aromatic rings, respectively [7,9].

Based on the FTIR analysis, PANI and its composites with citrate-, ascorbate-, and CTAB- capped AuNPs have the characteristic properties of the emeraldine form of polyaniline, whereas GSH-AuNP/PANI composite exhibits the properties of both, the fully oxidized pernigraniline form as well as emeraldine form of PANI [3,5]. The bluntness or absence of peaks at 1077, 1233, and 1291–1295 cm^−1^ along with the lowest intensity of N-H stretching at 1124 cm^−1^ can be accredited to the pernigraniline form in PANI composite with GSH-AuNPs [26,27]. The intensity of the peaks at 1475–1480 (peak A) and 1560 cm^−1^ (peak B) serves as a proxy for the relative content of benzenoid and quinoid ring, respectively. The A/B ratio is useful in assessing the conjugation length along the molecular chain in PANI [20,24]. Based on the FTIR spectral analysis, the A/B ratio in descending order was found to be PANI followed by its AuNP composites functionalized with CTAB, then ascorbate, citrate, and finally GSH. The differences in the ratio of peak A/peak B confirmed the variations in conjugation length order along the molecular chain of PANI in each developed material. The most interesting outcome achieved from the FTIR data (Table S1) is the unique characteristics of the GSH-AuNP/PANI. The GSH-AuNP/PANI composite has co-existence of both emaraldine and pernigraniline forms of PANI.

### 3.3. FE-SEM and HRTEM Based Morphology Studies

**Figure 3:**
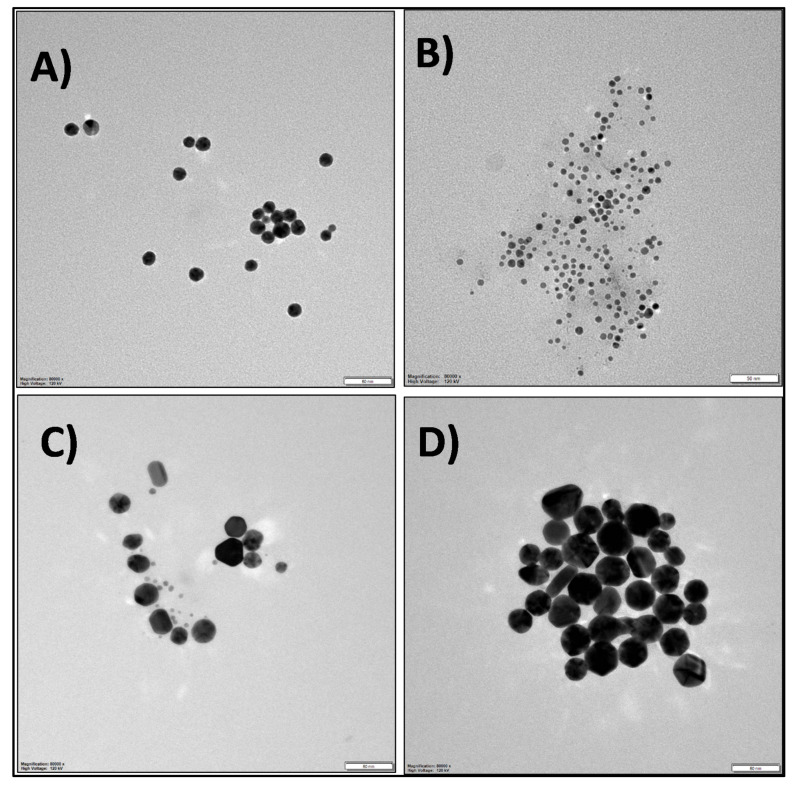
TEM images of AuNPs capped with different functional groups: (**A**) citrate, (**B**) ascorbate, (**C**) GSH, and (**D**) CTAB-functionalized AuNPs at 50 nm scale bar.

The TEM analysis of synthesized AuNPs and AuNP/PANI composites was performed to assess their particle size distribution and morphologies. The TEM images for citrate, ascorbate, GSH, and CTAB-capped AuNPs are shown in Figure 3. The corresponding average particle sizes of the capped AuNPs were obtained as 16.34, 8, 22.4, and 36.4 nm, respectively. Note that the average particle and fiber sizes were calculated using the “ImageJ” software. The zeta potential of the citrate-, ascorbate-, GSH-, and CTAB-capped AuNPs showcased their stability with the values of −43.2, −20.7, −27.8, and 22.8 mV, respectively (Figure S1).

**Figure 4:**
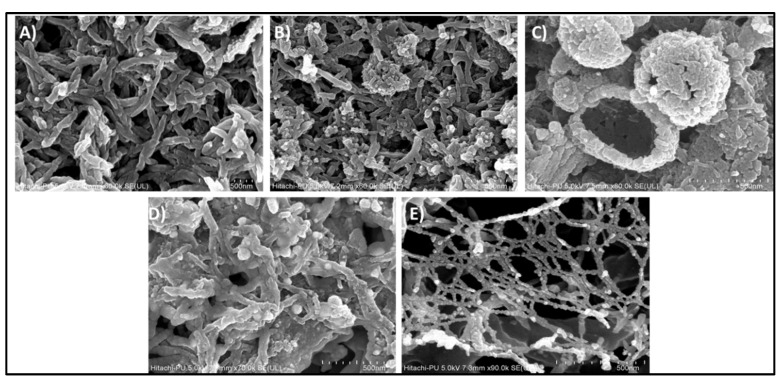
FE-SEM images (at 500 nm scale bar) of PANI and PANI/AuNPs composite samples prepared by interfacial polymerization with different types of functionalized AuNPs: (**A**) PANI, (**B**) citrate-AuNP/PANI, (**C**) ascorbate-AuNP/PANI, (**D**) GSH-AuNP/PANI, and (**E**) CTAB-AuNP/PANI.

**Figure 5:**
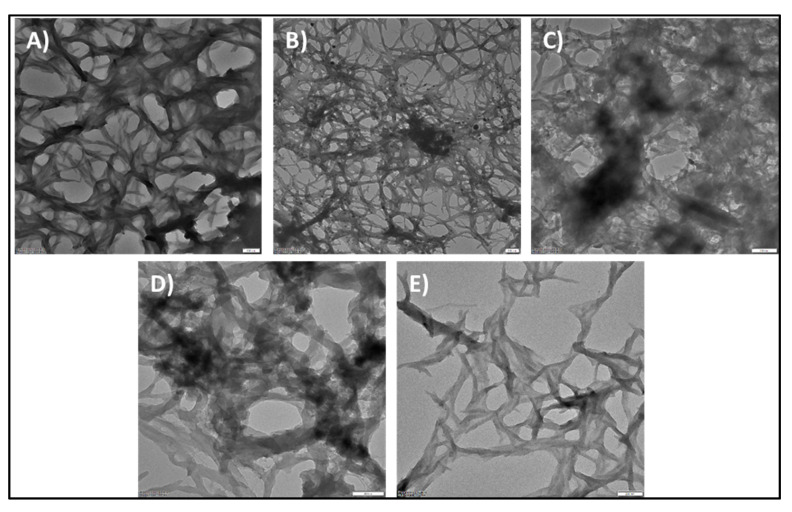
TEM images (at 200 nm scale) of PANI and PANI/AuNPs composite samples prepared by interfacial polymerization with different types of functionalized AuNPs: (**A**) PANI, (**B**) citrate-AuNP/PANI, (**C**) ascorbate-AuNP/PANI, (**D**) GSH-AuNP/PANI, and (**E**) CTAB-AuNP/PANI.

The surface morphologies of PANI and its AuNP composites were studied using FE-SEM (Figure 4), whereas internal architecture/structural distribution was analyzed using TEM (Figure 5). As seen in Figure 4, each type of materials fabricated in this study evidently exhibited their unique morphologies. The pure form of PANI exhibited elongated and uniform fibers. Moreover, as shown in Figures Figure 4A and Figure 5A, the obtained PANI fibers with average size of 71 nm displayed a random orientation in 3-D space. The detailed synthetic mechanism for PANI can be accessed from our earlier work [8]. In brief, upon the formation of interface between organic and aqueous phase, aniline monomers migrated into aqueous phase to form anilinium ions. These ions then served as nucleation centers to make the polymer grow further. AuNPs can also serve as nucleation centers for the growth of PANI, as their surfaces can favorably accommodate anilinium ions or PANI oligomers. The images of reaction vessel are provided to display the synthesis progression of PANI and AuNP/PANI composites in Figure S3.

**Figure 6:**
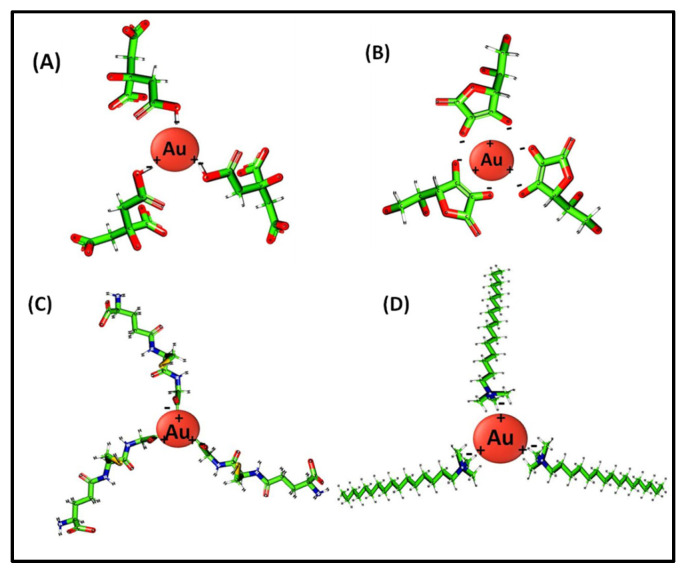
Modeled chemical structures of the capping agents used to functionalize AuNPs: (**A**) Citrate, (**B**) Ascorbate, (**C**) GSH, and (**D**) CTAB.

In case of AuNP/PANI composite forms, the morphology of the fibers obtained was heavily dependent on the type of AuNP functionalization and size of AuNPs. PANI synthesized in the presence of citrate- and ascorbate-functionalized AuNPs display nodular and hollow hemispherical vesicle-like structures, respectively. As is evident from Figure 4B and Figure 5B, the citrate- AuNP/PANI composites are arranged in the form of nodules (with diameter 313 nm) incorporating the PANI fibers (with diameter 40 nm). The centers of the nodules are expected to serve as nucleation centers for PANI growth while the over-growth of PANI led to the formation of nodule-like structures near nucleation centers. The nodule-like morphology in citrate-AuNP/PANI composites might be due to the interaction of anilinium ions with the three free negatively charged carboxyl-groups of the citrate on the AuNPs (Figure 6A) [28]. These groups are available simultaneously for the attachment of anilinium ions. It is suspected that the concurrent growth of polyaniline (after the attachment of anilinium ions) could lead to the formation of nodule-like structures.

In ascorbate-AuNP/PANI composite, the structures are arranged in large hollow hemispherical vesicles (550 nm) (Figure 4C and Figure 5C). The hollow cavity is visible in approximately 50% of the ascorbate-AuNP/PANI vesicles. The other 50% of the vesicles are either inverted/complete hollow or solid spheres. Moreover, these spherical structures are ~60% of the total ascorbate-AuNP/PANI composite structures visible in Figure S4. The small size of ascorbate-AuNPs (~8 nm) has restricted the average size of fiber below 28 nm. Additionally, the formation of hollow hemispherical vesicles can be expected due to the presence of ascorbate functionalization on AuNPs-surface. During the ascorbate-AuNPs synthesis, the –OH group on furan ring interacts with AuNP whereas the negatively charged aliphatic chain is free to contribute to negative charge of ascorbate-AuNPs [28]. Thus, ascorbate-AuNPs can directly interact with positively charged anilinium ions (see Figure 6B). The arrangement type of groups in ascorbate could facilitate the directional growth of the polyaniline fiber in the form of vesicles. Likewise, the PANI composites of AuNPs functionalized with GSH and CTAB are arranged in flakes and spider-web galaxy-like structures, respectively. The GSH-AuNP/PANI flakes with average size of 76 nm are assembled in tightly packed manner (Figure 4D and Figure 5D). The interactions of tripeptides (cysteine, glycine, and glutamate) present in GSH structure (Figure 6C) could be postulated as the origin for this flake-like morphology of GSH-AuNP/PANI composite. For example, cysteine can form disulfide linkages (-S-S-) with PANI. The ammonium persulfate used during the synthesis of PANI may serve as the source of sulfur in PANI to form –S–S– bond with GSH. Moreover, the scattered and negatively charged free carboxylic groups of the GSH would have participated in the growth of PANI to form sheet-like structure. This type of interaction between GSH-AuNPs and PANI can lead to the formation of a broad fiber with a flake-like appearance. On the other hand, the CTAB-AuNP/PANI composite shows loosely packed long fibers with uniform mesh (average size of 21 nm) which is comparable to the spider-web galaxy. These fibers are also guise-like separated beads (of 20 nm in size) on a thread as one may find in a necklace. The loose packing of CTAB-AuNP/PANI may be due to the presence of long aliphatic chains of the CTAB molecule capped on AuNPs (Figure 6D). This aliphatic chain can show π–π interaction with the anilinium ion and aniline oligomers to grow the polymer along the axis of aliphatic chain with the spider-web-like structures. The CTAB-AuNP/PANI fibers are arranged in a mesh-like structure, which could be helpful in imparting high porosity to this structure.

Overall, the incorporation of AuNPs with PANI not only decreased the size of the fibers but also controlled other morphological and/or electronic structures. The AuNPs were seen to serve as nucleation centers for the growth of PANI NFs. The AuNPs with different functionalities are thus speculated to drive the formation of diversely shaped AuNP/PANI composites with correspondingly diverse physical and chemical properties.

### 3.4. Electrochemical Properties of PANI and its Composites.

The electrochemical behavior of the synthesized PANI and AuNP/PANI composites was studied using cyclic voltammetry (CV).

**Figure 7:**
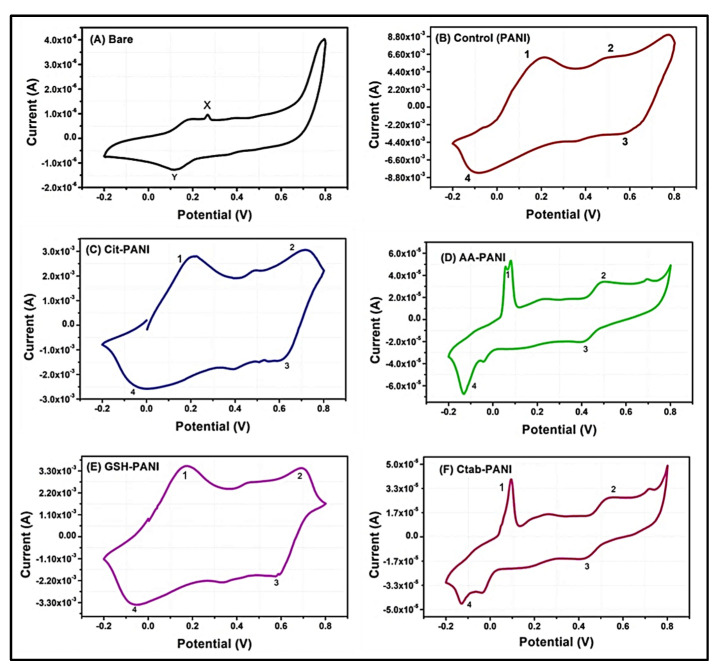
The electrochemical properties as studied using cyclic voltammetry on Au SPE: (**A**) bare electrode, (**B**) control (PANI), (**C**) citrate-AuNP/PANI, (**D**) ascorbate-AuNP/PANI, (**E**) GSH-AuNP/PANI, and (**F**) CTAB-AuNP/PANI. All the spectra were recorded with 0.5 M H_2_SO_4_ as electrolyte. X and Y represent the characteristic signals for the electrolyte. Peak labels 1, 2, 3, and 4 are transition peaks for emeraldine to pernigraniline (emer-perni), pernigraniline to emeraldine (perni-emer), emeraldine to leucoemeraldine (emer-leuco), and leucoemeraldine to emeraldine (leuco-emer) forms of PANI, respectively.

As shown in Figure 7A, the voltammogram of bare Au-SPE exhibited the signals for the electrolyte (H_2_SO_4_) near +0.2 V. As the same Au-SPE and electrolyte were used in every experiment, these peaks (X and Y) were the part of voltammograms recorded for the PANI as well as AuNP/PANI composites. Four peaks were observed in the voltammogram of PANI and AuNP/PANI composites responsible for the transition between the various forms of PANI. Specifically, the reversible transition of emeraldine form of PANI into pernigraniline form is represented by the redox signals as peaks 1 and 2 are present around +0.5 V, whereas the redox transition between emeraldine and leucoemeraldine forms (as represented by peak 3 and 4) is near −0.1 and 0.1 V, respectively [29].

Interestingly, the CV voltammograms for each composite have shown significant differences in terms of the potentio-dynamic behavior of the above mentioned conversion peaks in PANI composites. In case of PANI (Figure 7B), the transition peaks of 1 and 2 are recorded at +0.48 and +0.58 V, respectively. The transitions peaks of 3 and 4 were recorded at −0.06 and +0.19 V, respectively. In contrast, a different trend was observed in the transition peak potentials of the synthesized AuNP/PANI composites. In case of citrate-AuNP/PANI (Figure 7C), the transition peak potentials for 1, 2, 3, and 4 were observed at +0.70, +0.60, −0.019, and +0.20 V, respectively. The values of peak potentials for this composite have shown a change of +0.22 V for transition 1 (i.e., emeraldine -pernigraniline transition) with respect to PANI. Also, a slight increase of 0.02 V in emeraldine-pernigraniline conversion potential was observed. However, the transition potentials for 3 and 4 did not show any significant change. Thus, it can be concluded that in case of citrate-AuNP/PANI, emeraldine form required enhanced potential for conversion into pernigraniline form relative to PANI alone. In contrast, in case of ascorbate-AuNP/PANI (Figure 7D), the transition signals show significant variations in peak potentials and current relative to control PANI. The potential of transition peaks 1, 2, 3, and 4 were observed at +0.47, +0.42, −0.12, and +0.54 V, respectively. This observation indicates the negligible change in the content of pernigraniline form of PANI, when compared to the PANI and citrate-AuNP/PANI. Moreover, a higher potential value of ~0.40 V was required for the redox conversion between emeraldine and leucoemeraldine forms of PANI. It can thus be concluded from current intensities (Figure 7D) that the higher amounts of leucoemeraldine form should readily be converted into the emeraldine and successively into pernigraniline forms upon redox conversions.

The GSH-AuNP/PANI (Figure 7E) composite has shown unique feature with almost equal transition signals for all the peaks (i.e., ~ 0.30 mA). The respective peak potentials for the transitions 1, 2, 3, and 4 were recorded at +0.68, +0.58, −0.53, and +0.16 V, respectively. These values of current and peak potential suggest that GSH-AuNP/PANI should favorably produce both emeraldine and pernigraniline forms of PANI while leucoemeraldine form is readily converted into the emeraldine form. In other words, it is thus expected that almost equal amounts of emeraldine and pernigraniline forms might be present in this composite. In case of CTAB-AuNP/PANI, the observed transition potentials for peak 1, 2, 3, and 4 were +0.52, +0.43, −0.12, and +0.93 V, respectively. The CTAB-AuNP/PANI displayed similar redox conversion pattern as that of ascorbate-AuNP/PANI other than a splitting of peak 3 in latter case. The occurrences of this peak split might reflect the effects of some artifacts on electrode surface. It was observed from the voltammogram of CTAB-AuNP/PANI that the emeraldine form was converted into high amounts of leucoemeraldine form, which was subsequently converted into the pernigranline form upon applying further voltage. In addition, for CTAB-AuNP/PANI, the signals for the transition between different forms of PANI were observed to be damped as compared to the other composite. This might probably occur due to the long aliphatic chain in the structure of the CTAB as capping agent [30].

Overall, as seen from the specific CV signatures of each AuNP/PANI composite and the PANI samples, the presence of different forms of PANI was validated with the accompanying changes in the electrochemical characteristics. The variations in transition peak potential for the inter-conversion of the three forms of PANI, as shown in Table S2, provide a good insight into the difference in the conductivity behaviors of the synthesized PANI composites. These unique and distinctive properties of AuNP/PANI are dependent mainly on the differentially capped AuNPs, which can be helpful in expanding their applications in the field of energy storage, drug delivery, and microelectronics.

### 3.5. Morphology Orientation of AuNP/PANI Composites and Comparative Evaluation with Other Synthesis Methodologies for PANI-Materials

This study was carried out to demonstrate the tremendous potential of PANI composites with AuNPs in that their morphological properties can be efficiently adjusted to enhance their versatile applicabilities. For instance, the nanofibrous forms of conductive PANI could take the advantages in the fabrication of electrochemical sensing and optoelectronic/electrical devices [31,32]. Likewise, the vesicle form of the PANI composite is a highly useful option as a carrier for molecules (such as drugs, enzymes, proteins, and pesticides), supercapacitor, sorbent, and catalyst [32,33,34]. PANI structures with spider web-like morphology and high porosity, on the other hand, are useful for sorbent and capacitor applications [35,36]. The nodule-like structures also exhibit excellent potential for diverse applications in electrochemical energy storage, pollutant removal, supercapacitors, and sensors [37,38,39,40].

Previous efforts have attempted to control the morphology of PANI composite [4]. The adsorption or assembly pattern of oligomers on nucleation centers was concluded to be primarily responsible for the growth of different morphologies. In this research, it was also observed that different capping of AuNPs (acting as nucleation centers) directed the growth of PANI in diverse morphologies. The nodule-like morphology was also obtained by keeping the interfacial synthesis temperature to 0 °C [41]. These researchers also tested the effect of several synthesis parameters (e.g., concentration of the aniline, amount of ammonium persulfate, and solvents for the development of PANI) on the morphological features of PANI. Nonetheless, they were only able to control the alignment while reducing the size of the fibers. Likewise, the effects of variables like reaction temperature, pH, and duration were also examined in relation to morphological forms of PANI [42,43]. These variations in the synthesis procedure were effective to yield diverse morphologies of PANI such as nanospheres and nanorods. ZnO, TiO_2_, and swollen liquid crystal (SLC) templates have also been explored for PANI morphologies of NFs (100 nm), nanowires (10 μm), and spheroids, respectively [44,45,46]. Similarly, the addition of varying concentrations of steric stabilizer, such as poly(N-vinylpyrrolidone) (PVP), can yield three different morphologies of PANI, i.e., nanospheres (50 nm), nanorods (150 nm), and NFs (215 nm) [47]. In a few other studies, the use of K_2_Cr_2_O_7_ and CTAB during chemical synthesis of PANI yielded petal-like and rice-like structures, respectively [20,48,49].

In the majority of the above-mentioned studies, the PANI structures formed were of much larger dimensions than the structures developed in this study. Moreover, only solvothermal synthesis methods were reported to generate morphologies other than fibrous structures of PANI-materials. We were able to attain diverse morphologies of PANI materials through interfacial polymerization. To expand their morphological characteristics, PANI-based materials can be prepared in a more controlled manner with interfacial polymerization [31]. As seen in this study, the structures of PANI composites were modified effectively through interfacial polymerization. Thus, this research demonstrates that the approaches introduced should benefit the development of high throughput technologies for PANI composites.

## 4. Conclusions

In this work, we prepared PANI and its AuNP composites with four different functionalities through interfacial polymerization. The obtained nanostructures exhibited high diversity in terms of morphological/chemical characteristics and electrochemical properties. A random NF morphology was obtained from PANI alone, while PANI-AuNP composites functionalized with citrate, ascorbate, GSH, and CTAB were capable of taking diverse morphologies such as nodule, hollow hemisphere, flake, and spider-web galaxy type, respectively. A fully oxidized pernigraniline form of PANI is estimated to be formed in case of GSH-AuNP/PANI composite, while PANI, citrate-AuNP/PANI, ascorbate-AuNP/PANI, and CTAB-AuNP/PANI should be present in emeraldine salt form. These results have all been validated with several visual and characterization methods such as color change, UV-Vis, FTIR, and electrochemical studies. In this study, some control on the physical (morphology) and electrical/electrochemical properties of PANI composites has been realized through interfacial polymerization. The results of this study thus suggest that the applicability of AuNP/PANI-based nanocomposites can be extended considerably for diverse technological applications in sensing, energy storage, drug delivery, and pollutant removal.

## Supplementary Materials

The following are available online at https://www.mdpi.com/2079-4991/10/12/2404/s1, Section S1: Zeta potential of differentially capped AuNPs, Section S2: Synthesis of PANI and AuNP/PANI composites, Section S3: Progression of PANI polymerization, Section S4: FTIR data analysis, Figure S1: The zeta potentials of the differently capped AuNPs incorporated in the composite formation with PANI: (A) Citrate-AuNPs, (B) Ascorbate-AuNPs, (C) GSH-AuNPs, and (D) CTAB-AuNPs, Figure S2: The images for the interfacial polymerization of PANI and its composites with AuNPs during progression of reaction: (A) PANI, (B) citrate-AuNPs/PANI, (C) CTAB-AuNPs/PANI, (D) GSH-AuNPs/PANI, and (E) ascorbate-AuNPs/PANI, Figure S3: Images for the interfacial polymerization of PANI and its composites with AuNPs during progression of reaction: (A) PANI, (B) citrate-AuNPs/PANI, (C) CTAB-AuNPs/PANI, (D) GSH-AuNPs/PANI, and (E) ascorbate-AuNPs/PANI, Figure S4: SEM image of ascorbate-AuNP/PANI composite to show the distribution of spherical vesicles (at 1 µm scale bar), Table S1: IR bands and their assignment in synthesized PANI and AuNP/PANI composites, Table S2: The values of transition peak potentials (measured by CV) for PANI and its composites.
