# Supplementary material for: A Novel Approach for Effective Alteration of Morphological Features of Polyaniline through Interfacial Polymerization for Versatile Applications
Source: Nanomaterials (Basel). 2020 Nov 30;10(12):2404. doi: 10.3390/nano10122404 (PMC7760113; doi:10.3390/nano10122404)
Supplement: Supplementary file 1 [file nanomaterials-10-02404-s001.pdf]

Supplementary Materials: A Novel Approach for Effective Alteration of Morphological Features of Polyaniline through Interfacial Polymerization for Versatile Applications

Kalyan Vaid [1,2,†], Jasmeen Dhiman [1,†], Suresh Kumar [3], Ki-Hyun Kim [4,*] and Vanish Kumar [1,*]

1. National Agri-Food Biotechnology Institute (NABI), S.A.S. Nagar, Punjab 140306, India; vaidkalyan@gmail.com (K.V.); jasmeendhiman10@gmail.com (J.D.)
2. Centre for Nanoscience and Nanotechnology, Panjab University, Chandigarh 160014, India
3. Department of Applied Sciences, UIET, Panjab University, Chandigarh 160014, India; skphysicsnano@gmail.com
4. Department of Civil and Environmental Engineering, Hanyang University, 222 Wangsimni-ro, Seoul 04763, Korea

* Correspondence: kkim61@hanyang.ac.kr (K.-H.K.); vanish@nabi.res.in (V.K.)

† These authors contributed equally to this work.

S1. Zeta potential of differentially capped gold nanoparticles (AuNPs)

The zeta potentials of the citrate-, ascorbate-, GSH-, and CTAB-capped AuNPs were measured as −43.2 mV, −20.7 mV, −27.8 mV, and 22.8 mV, respectively (**Figure S1**).

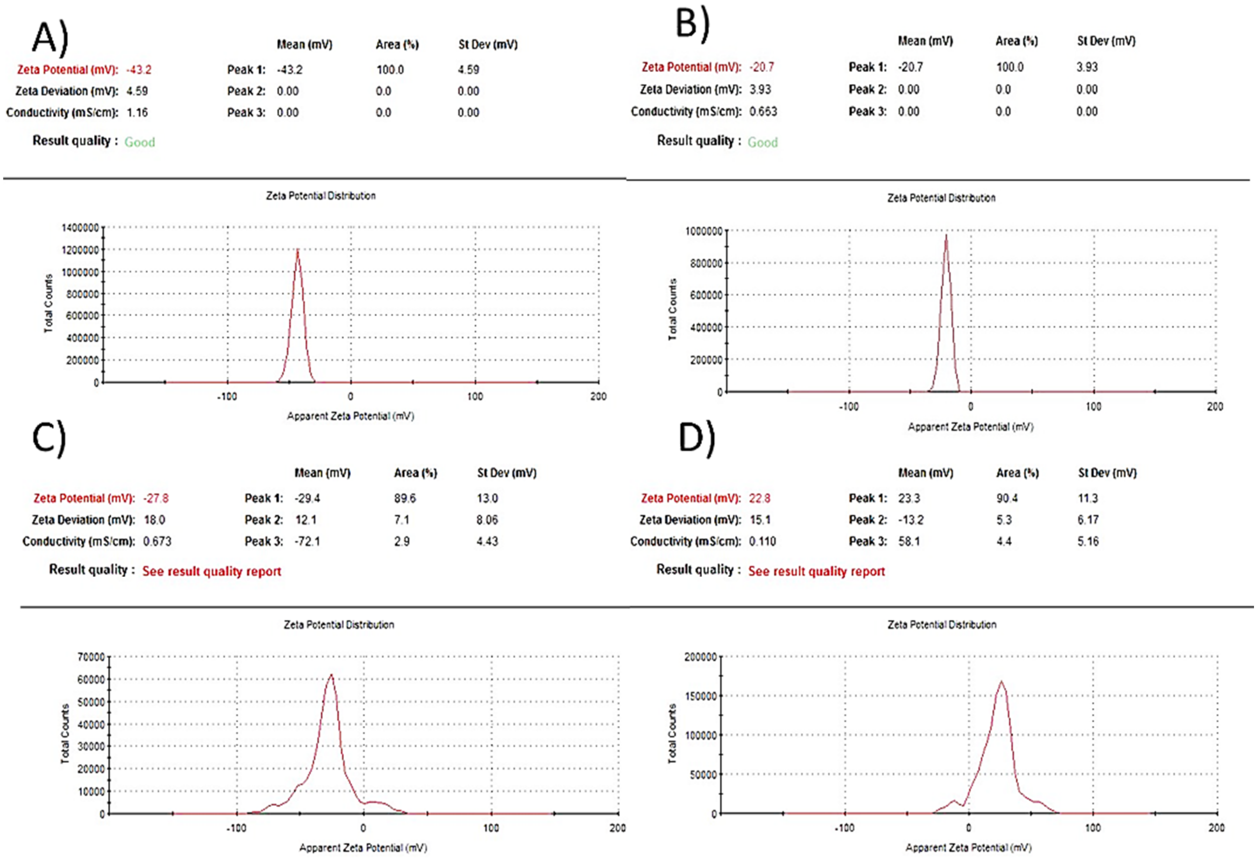

Figure S1. The zeta potentials of the differently capped AuNPs: **A)** Citrate-AuNPs, **B)** Ascorbate-AuNPs, **C)** GSH-AuNPs, and **D)** CTAB-AuNPs.

S2. Synthesis of PANI and AuNP/PANI composites

After the formation of interface between aqueous and organic phase, the PANI and PANI composites with -citrate, -ascorbate, -CTAB, and GSH-capped AuNPs started to develop in aqueous phase. It is worth mentioning here that the green colored product was formed for PANI, citrate-AuNP/PANI, ascorbate-AuNP/PANI, and CTAB-AuNPs/PANI (indicating formation of emeraldine form of PANI). In contrast, brown colored material was obtained in case of GSH-AuNP/PANI (indicating pernigraniline form of PANI).

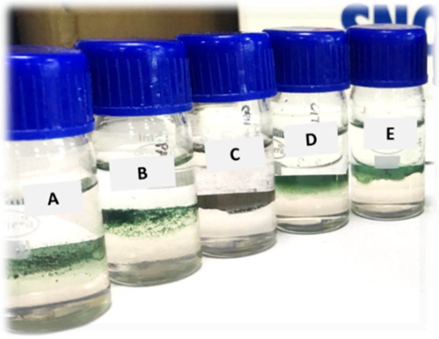

Figure S2. Images of reaction containers consisting of PANI and AuNP/PANI composites in aqueous phase: **A**) PANI, **B**) citrate-AuNP/PANI, **C**) GSH-AuNP/PANI, **D**) Ascorbate-AuNP/PANI, and **E**) CTAB-AuNPs/PANI.

S3. Progression of PANI polymerization

The images of reaction vessels to display the progression of PANI and AuNP/PANI composites synthesis are shown in **Figure S3**.

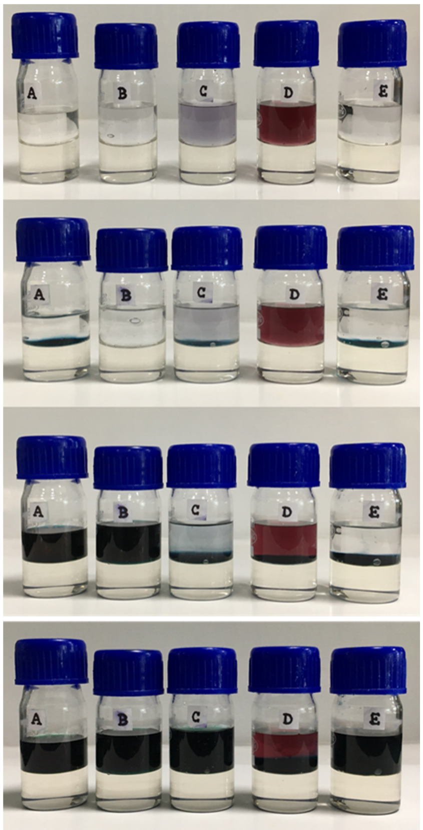

Figure S3. Images for the interfacial polymerization of PANI and its composites with AuNPs during progression of reaction: **A**) PANI, **B**) citrate-AuNPs/PANI, **C**) CTAB-AuNPs/PANI, **D**) GSH-AuNPs/PANI, and **E**) ascorbate-AuNPs/PANI.

S4. FTIR data analysis

Table S1. IR bands and their assignment in synthesized PANI and AuNP/PANI composites.

Sr No.	Wavenumber (cm^{-1})	Functional group assignment	Synthesized PANI-materials				
			PANI	Citrate-AuNP/PANI	Ascorbate-AuNP/PANI	GSH-AuNP/PANI	CTAB-AuNP/PANI
1.	1560	C=C stretching of quinoid ring polaronic structures (–B–NH^+–)	Present	Present	Present	Present	Present
2.	1475–1480	C=C stretching of benzenoid ring in –NH–B–NH– units	Present	Present	Present	Present	Present
3.	1400	Phenazine type segments	Present	Present	Present	Absent	Absent
4.	1291–1295	C–H stretching of aromatic amine	Present	Present	Present	Present	Present
5.	1233	C–N stretching vibrations in benzenoid unit	Present	Present	Present	Present blunt form	Present
6.	1124	N–H stretching	Present	Present	Present	Present but with very low intensity	Present
7.	1026	In-plane bending of C–H of aromatic rings	Present	Present	Present	Present	Present
8.	730	Imine deformation (C–N–C bending)	Present	Present	Present	Present	Present
9.	799	Quinone ring deformation	Present	Present	Present	Present	Present
10.	600	In-plane deformation vibrations of aniline groups of bipolaronic structure	Present	Present	Present	Present	Present

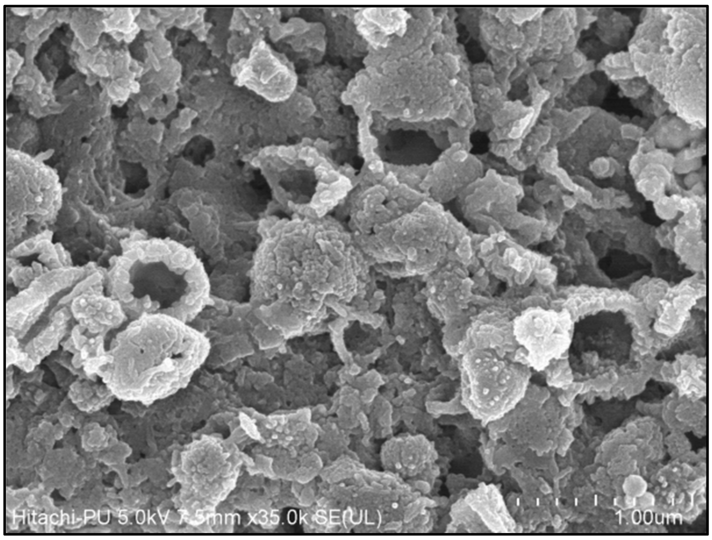

Figure 4. SEM image of ascorbate-AuNP/PANI composite to show the distribution of spherical vesicles (at 1 μm scale bar).

Table 2. The values of transition peak potentials (measured by CV) for PANI and its composites.

	**Transition Peak Potential (V)**			
	1<br>(Emeraldine to Pernigraniline)	2<br>(Pernigraniline to Emeraldine)	3<br>(Emeraldine to leucoemeraldine)	4<br>(Leucoemeraldine to Emeraldine)
PANI	+0.48	+0.58	−0.06	+0.19
Citrate-AuNP/PANI	+0.70	+0.60	−0.019	+0.20
Shift w.r.t. PANI	+0.22	+0.02	−0.041	+0.01
Ascorbate-AuNP/PANI	+0.47	+0.42	−0.12	+0.54
Shift w.r.t. PANI	+0.01	−0.16	+0.06	+0.35
GSH-AuNP/PANI	+0.68	+0.58	−0.53	+0.16
Shift w.r.t. PANI	+0.20	0	+0.47	+0.03
CTAB-AuNP/PANI	+0.52	+0.43	−0.12	+0.93
Shift w.r.t. PANI	+0.04	−0.15	−0.12	+0.72
